# A close look at protein function prediction evaluation protocols

**DOI:** 10.1186/s13742-015-0082-5

**Published:** 2015-09-14

**Authors:** Indika Kahanda, Christopher S Funk, Fahad Ullah, Karin M Verspoor, Asa Ben-Hur

**Affiliations:** 1 Department of Computer Science, Colorado State University, Fort Collins, 80523 CO USA; 2 Computational Bioscience Program, University of Colorado School of Medicine, Aurora, 80045 CO USA; 3 Department of Computing and Information Systems, University of Melbourne, 3010 Parkville, Victoria, Australia

**Keywords:** Automated function prediction, Gene Ontology, Machine learning, Support vector machines

## Abstract

**Background:**

The recently held Critical Assessment of Function Annotation challenge (CAFA2) required its participants to submit predictions for a large number of target proteins regardless of whether they have previous annotations or not. This is in contrast to the original CAFA challenge in which participants were asked to submit predictions for proteins with no existing annotations. The CAFA2 task is more realistic, in that it more closely mimics the accumulation of annotations over time. In this study we compare these tasks in terms of their difficulty, and determine whether cross-validation provides a good estimate of performance.

**Results:**

The CAFA2 task is a combination of two subtasks: making predictions on annotated proteins and making predictions on previously unannotated proteins. In this study we analyze the performance of several function prediction methods in these two scenarios. Our results show that several methods (structured support vector machine, binary support vector machines and guilt-by-association methods) do not usually achieve the same level of accuracy on these two tasks as that achieved by cross-validation, and that predicting novel annotations for previously annotated proteins is a harder problem than predicting annotations for uncharacterized proteins. We also find that different methods have different performance characteristics in these tasks, and that cross-validation is not adequate at estimating performance and ranking methods.

**Conclusions:**

These results have implications for the design of computational experiments in the area of automated function prediction and can provide useful insight for the understanding and design of future CAFA competitions.

**Electronic supplementary material:**

The online version of this article (doi:10.1186/s13742-015-0082-5) contains supplementary material, which is available to authorized users.

## Background

Proteins are the workhorses of life, and identifying their functions is an important biological problem. The Gene Ontology (GO) [1] is a structured vocabulary that captures protein function in a hierarchical manner. Through various wet-laboratory experiments over the years, scientists have been able to annotate a large number of proteins with GO categories that reflect their functionality. However, experimentally determining protein functions is a highly resource-consuming task. The reasonable success in computationally determining the functions of proteins using a variety of data sources–by homology from sequence/structure, using various biological network data, and by text mining [2–5]–has led to automated function prediction (AFP) being established as an important problem in bioinformatics.

As a result of the emergence of a multitude of computational methods for protein function prediction, the community has realized the need for a systematic and organized means of comparing the performance of these methods so as to assess how far the area has progressed. Taking note from critical assessment efforts such as Critical Assessment of protein Structure Prediction (CASP) [6] and Critical Assessment of Prediction of Interactions (CAPRI) [7], the AFP community decided to hold its own competition: Critical Assessment of Function Annotation (CAFA) [5]. The main objective of CAFA is to gather all AFP researchers in one place to fairly assess and compare the latest computational methods using a centralized and independent assessment. In the first CAFA (CAFA1) the participants were provided with a list of protein targets that did not have any previous annotations and were asked to submit computational predictions using their own AFP methods [5]. Once the predictions were submitted, the organizers collected the experimentally validated GO annotations acquired for the targets over a period of 6 months. Finally the computational predictions were compared against those annotations to compute the accuracy of each AFP method.

The recent CAFA2 challenge [8] had exactly the same setup, except that the list of 100,000 target proteins consisted of both annotated and unannotated proteins. The added requirement of making predictions on currently annotated proteins makes CAFA2 a more realistic representation of the function prediction problem, as it better models the accumulation of annotations over time. We identify the CAFA2 requirements as a combination of two subtasks: making predictions on annotated proteins and making predictions on unannotated proteins.

The AFP problem posed in CAFA is more challenging than the typical machine learning problem, as the usual assumption in machine learning is that the distribution of examples in the training set is reflective of that in the test set. In the CAFA AFP problem this assumption probably does not hold because the training is performed on an older set of annotations whereas testing is performed on newer annotations; and it is known that the distribution of GO categories changes over time as a result of strong biases in the annotation process [9]. Furthermore, the annotations acquired for annotated proteins and for unannotated proteins can be expected to differ in frequency and specificity: an annotated protein can be expected to acquire more specific GO categories than an unannotated protein, and perhaps more of them, as the biology community tends to study proteins that are already characterized. The COMBREX project is an effort to address this bias [10].

In this study we clearly delineate the differences between these two AFP tasks (i.e. the task of making predictions for annotated proteins and the task of making predictions for unannotated proteins) and how different AFP methods perform in each case. We also compare the performance of AFP methods on these tasks with their performance in cross-validation (CV), which is typically used by many to assess and compare AFP methods. Finally, we determine whether the performance metric has an impact on the ranking of different methods. Our results have ramifications for practitioners in the area of AFP, which should help them in the design of their computational experiments.

## Data Description

### Prediction tasks

We identify two tasks in the area of AFP: prediction of annotations for proteins without previous annotations (denoted ‘novel proteins’ or NP) and prediction of novel annotations for proteins that already have some annotations associated with them (denoted ‘novel annotations’ or NA). To understand the relative difficulty of these tasks we compare the performance of several AFP methods under evaluation protocols that directly capture these tasks and also compare them to the typically used alternative, which is CV. In what follows we describe in detail the protocols, the data used, and the algorithms that were compared.

### Evaluation protocols

We compare three experimental setups: CV, NA and NP. CV is the standard CV setup that is typically used for evaluating AFP methods; more specifically, we use 5-fold CV in which each fold corresponds to a randomly chosen set of proteins.

**Fig. 1:**
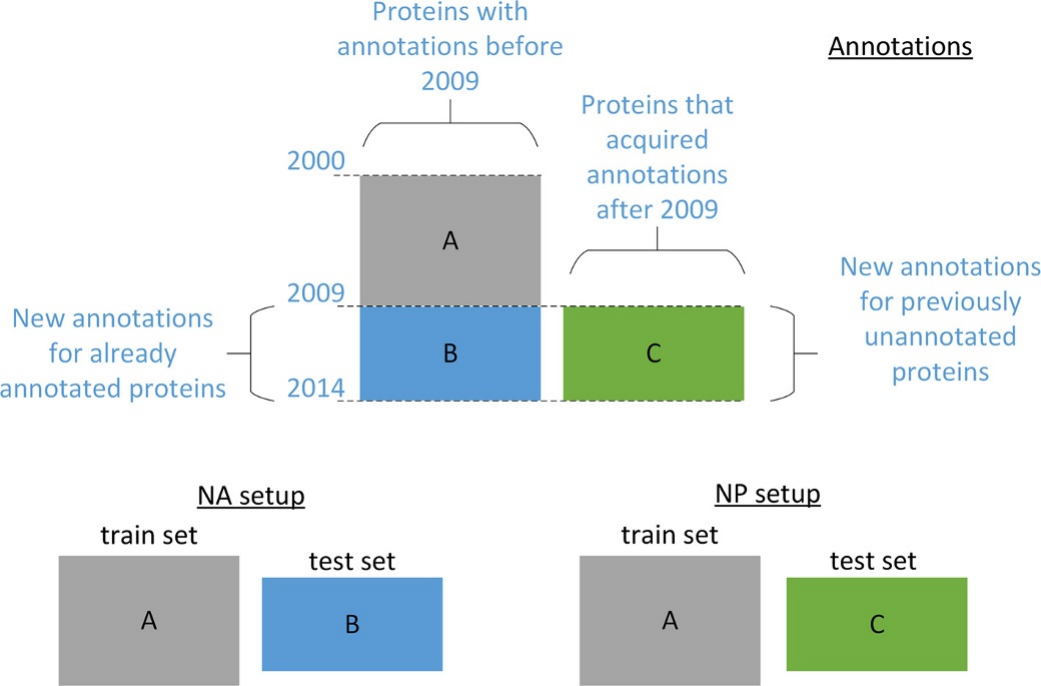
Overview of the NA and NP setups. We distinguish between three sets of annotations that are used to define the training and test set in the two setups. Annotations accumulated between an initial time *t*_0_ until *t*_1_ (end of 2009 in our experiments) and form a set A, which is the training set in both NA and NP. The set of annotations acquired for those proteins after *t*_1_ form a set B, which is the test set in the NA setup. The set of annotations acquired after *t*_1_ for proteins that were unannotated before *t*_1_ is denoted by the set C, and is used as the test set in the NP setup

In the NA protocol, methods are trained using the set of annotations acquired in or before the year 2009, and tested on the set of annotations gathered on the same set of proteins after 2009 (the ‘GO annotations’ section below fexplains the criteria used for selecting the final set of annotations). In other words, the same set of proteins are used for training and testing, but the training labels are annotations that were available in 2009, whereas testing labels are annotations made available after 2009. An annotation that was added with a new evidence code but is present in the training set was not included in testing; similarly, we do not include an annotation in the test set when a more specific annotation already exists in the training set. In the NP protocol methods are trained using the set of annotations acquired in or before the year 2009 and they are tested on the annotations acquired for proteins that were not annotated in or before 2009. In this setup, the proteins used for training and the proteins used for testing are disjoint sets. An overview of the NA and NP setups is given in Fig. 1.

In our experiments we focused on yeast and human; the number of proteins/annotations in the training/test sets with respect to the three setups are given in Tables 1 and 2. We note that our yeast/human test sets contain 5–10 times more proteins/annotations for each species and subontology combination than in the CAFA1 and CAFA2 challenges: In CAFA1 the test set consisted of 866 targets across 11 species consisting of five yeast proteins (one with molecular function and five with biological process annotations) and 285 human proteins (182 with molecular function and 195 with biological process annotations) [5]. In the information made available during the CAFA2 workshop the organizers revealed that CAFA2 test sets were composed of 656, 773 and 991 proteins with molecular function, biological process and cellular component annotations, respectively. These come from 27 species, but the vast majority of them were human proteins. The large number of annotations used here allowed us to compute term-centric metrics in addition to the protein-centric metrics used in CAFA.

**Table 1:** The number of proteins and the number of annotations in the train and test sets with respect to the three setups for yeast

		Training set		Test set	
	Setup	Proteins	Annots.	Proteins	Annots.
F	CV	1532	2185	383	546
	NA	1367	1706	208	285
	NP	1367	1706	521	677
P	CV	2752	5789	688	1447
	NA	2834	5161	633	990
	NP	2834	5161	604	1046
C	CV	3731	7053	932	1763
	NA	4189	6968	813	1162
	NP	4189	6968	476	681

F, P and C represent molecular function, biological process and cellular component, respectively. For the CV setup, numbers represent average values computed across the training and test folds (5-fold CV)

**Table 2:** The number of proteins and the number of annotations in the train and test sets with respect to the three setups for human

		Training set		Test set	
	Set.	Proteins	Annots.	Proteins	Annots.
F	CV	4532	8467	1133	2116
	NA	4305	6898	799	1343
	NP	4305	6898	1344	2174
P	CV	7533	31794	1883	7948
	NA	5824	12196	3301	13192
	NP	5824	12196	3574	12973
C	CV	8440	19196	2110	4799
	NA	5082	8185	2966	5511
	NP	5082	8185	5468	10200

F, P and C represent molecular function, biological process and cellular component, respectively. For the CV setup, numbers represent average values computed across the training and test folds (5-fold CV)

To perform a fair comparison across setups we first identified the GO subgraph that consists of only the GO categories common to all three setups (CV, NA and NP). Then we computed the evaluation measures described next only on this subgraph.

## Analyses

**Fig. 2:**
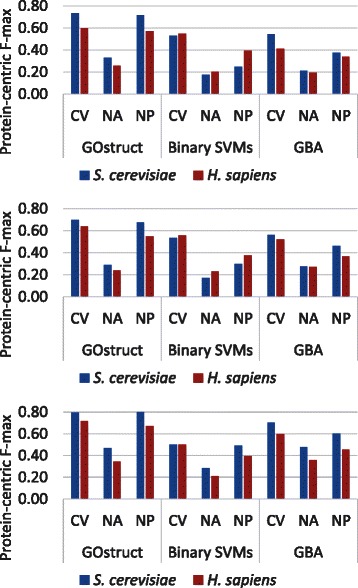
Performance comparison between CV, NA and NP. GOstruct, binary SVMs and GBA are evaluated in cross-validation (CV), novel annotation (NA) and novel proteins (NP) in yeast and human. The top, middle and bottom panels depict the molecular function, biological process and cellular component subontologies, respectively. Performance is evaluated using the protein-centric F-max

To evaluate how different AFP methods perform on the NA and NP tasks, and how these evaluation protocols compare with CV, we compared the performance of GOstruct, binary SVMs and Guilt by Association (GBA) using the three evaluation setups (CV, NA and NP) using data from yeast and human. The results for the protein-centric F-max performance measure are shown in Fig. 2 (see Methods for precise definition of F-max and the other performance measures). Additionally, *p*-values computed using paired t-tests for the differences in performance between CV and NA/NP are given in Tables S1 and S2 in Additional file 1. It can be observed that accuracy computed using CV is much higher than in the NA and NP protocols in both human and yeast and across all three GO hierarchies. This difference is even more pronounced when using the term-centric F-max measure (see Figures S1-S3 in Additional file 1). The only exception to this trend is the similar performance of GOstruct in the NP protocol, as discussed in more detail below. These results suggest that in most cases CV is not a good proxy for the performance in the more realistic NA and NP protocols.

The observed difference in performance between CV and the NA and NP protocols can be traced to the evolution of GO curation. It is known in the AFP community that the process of acquiring GO annotations is highly biased, leading to a distribution of categories that changes over time [9]. Because machine learning methods rely on the assumption that the distribution of test examples is the same as the training examples, this bias makes the NA and NP tasks more difficult than performing well in CV. Given that CV mixes annotations across time, the training and test sets are more similar in terms of the categories annotated, and performance can therefore be expected to be higher.

**Fig. 3:**
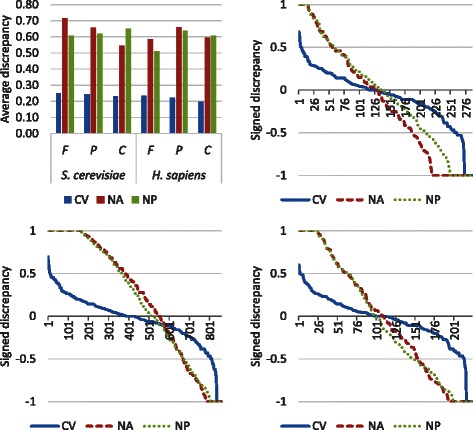
Label distribution comparison between CV, NA and NP. First we computed the probability (number of annotated proteins/number of all proteins) of GO category *i* in the training and test sets for all three setups, denoted by $p_{i}^{\text {tr}}$ and $p_{i}^{\text {tst}}$, respectively; in the CV setup the calculation was performed five times for each fold and averaged across the five folds. The discrepancy for category *i* is then defined as: $|p_{i}^{\text {tr}} - p_{i}^{\text {tst}}| / (p_{i}^{\text {tr}} + p_{i}^{\text {tst}})$. The average discrepancy is shown in top left panel. *p*-values based on paired t-tests for CV vs NA and CV vs NP in all three subontologies for both species are less than 1E−4 or 10^−4^. The individual signed discrepancy values (without the absolute value) are shown in the other three panels in sorted order by their magnitude for each setup

To demonstrate the differences in the label distributions in the training set versus the test set, we performed the following analysis. First we computed the probability (number of annotated proteins/total number of proteins) of GO category *i* in the training and test sets for all three setups, denoted by $p_{i}^{\text {tr}}$ and $p_{i}^{\text {tst}}$, respectively; in CV setup the calculation was performed five times for each fold and averaged across the five folds. The discrepancy for category *i* is then defined as: $|p_{i}^{\text {tr}} - p_{i}^{\text {tst}}| / (p_{i}^{\text {tr}} + p_{i}^{\text {tst}})$. The mean discrepancy and individual signed discrepancy values (without the absolute value) are shown in Fig. 3 and Figures S4-S6 in Additional file 1. We observe that the average discrepancy for NA and NP is significantly higher than in the CV setup in the three subontologies for both yeast and human, suggesting that the label distributions between training and test sets in NA and NP is consistently different from that in CV.

We hypothesize that this characteristic is at least partly responsible for the lower performance in the NA and NP setups. To provide evidence for this hypothesis, we computed the correlation between area under the receiver operating characteristic curve (AUC) scores and the discrepancy values for each GO category. As illustrated in Figure S7 in Additional file 1, the AUC scores are negatively correlated with the discrepancy values, suggesting that for GO categories that show a larger difference between the training and test probabilities the performance tends to be lower. This is more pronounced for NA than NP, which is in agreement with our observation of lower accuracy for the NA protocol, as discussed below.

To further explore the differences between the setups, we considered the observation of Gillis and Pavlidis [28] that node degree, which is an indicator of multi-functionality, is highly correlated with classifier accuracy as measured by CV. We ran a similar experiment: for each setup we computed the correlation between each of the three classifiers’ AUC scores across GO terms in the biological process subontology with a simple classifier that predicts each GO term with a confidence given by the corresponding gene’s degree. The highest correlations were observed in the CV setup, followed by NP and NA (see Table S3 in Additional file 1); in the NA setup some of the correlations were insignificant, and even negative. This suggests that in the NA setup, the classifier cannot make use of information about node degree as well as in the other setups, explaining its lower performance. We also observe significant correlations between the performance of the node degree classifier and the discrepancy in term prevalence (Figure S8 in Additional file 1). Another factor worth exploring is the presence of critical edges, which are edges whose removal has a strong effect on classifier performance [27].

It is also interesting to note that the key observation of NA and NP performance being lower than CV is not due to a small-sample effect. As reported in Tables S4 and S5 in Additional file 1, by performing the evaluation only on the well-represented GO categories with 50 or more annotations, we see the same patterns as the evaluation on all GO categories.

Another observation is that the ranking of methods on the basis of CV performance is not the same as that which is obtained using the other protocols. The protein-centric F-max of binary SVMs (0.55) is higher than that of GBA (0.41) in CV in the molecular function subontology in human, but its performance on the NA task is equal to that of GBA (0.20) (Fig. 2); the protein-centric F-max of binary SVMs (0.53) is very similar to that of GBA (0.54) in CV in the molecular function subontology in yeast, but its performance in the NP task (0.25) is much lower than that of GBA (0.37) (Fig. 2). These observations suggest that a ranking of methods established using CV may not reflect how they would rank on other AFP tasks, which has implications for the design of method evaluation in AFP.

Among the three protocols, NA yielded the lowest accuracy for all methods, that is, the task of predicting novel annotations for previously annotated proteins is harder than prediction of novel annotations for unannotated proteins.

There are several reasons why NA is harder than NP. First, unlike the NP protocol, the NA evaluation protocol uses only the annotations acquired after 2009 as the ground truth. This means that a small mistake in NA has a larger impact on accuracy than what it would in NP. See Figure S9 in Additional file 1 for a specific example that illustrates this phenomenon. Second, our intuition suggests that in the beginning a protein is annotated with GO categories that are less specific or easier to experimentally verify (i.e. low-hanging fruit), and as time goes on, with the improvement of experimental assays, more specific annotations are added. We believe this is responsible for making the NA problem harder than NP. For the GOstruct method the difference between CV and NA accuracy is the most pronounced, although it is still the best-performing method.

When it comes to the NP task, we observe different trends across methods. In this task, GOstruct performs almost equally well as in CV, as can be seen in Fig. 2, whereas all the other methods show a much bigger decline in performance. This can be attributed to the fact that GOstruct uses the set of all combinations of GO categories in the training set as its set of candidate labels. As a result, it is likely that when predicting on currently unannotated proteins those candidate labels more closely represent the GO category combinations that are expected to be annotated in these new proteins. The two other binary classifiers, binary SVMs and GBA, do not use this information, and they struggle to achieve the same level of performance as in CV. But their performance in the NP task is still always better than in the NA task.

Our final observation is that the ranking of methods varies between evaluation metrics. We compared performance measured using protein-centric F-max, which was used in the CAFA competitions, with performance measured using the term-centric F-max (complete results using term-centric F-max and term-centric AUC are shown in Tables S6 and S7 in Additional file 1. The term-centric F-max of binary SVMs (0.12) is significantly higher than that of GBA on the NA task (0.08) in the molecular function subontology in human (*p* value: 7.03E-07 based on paired t-test; see Table S7 in Additional file 1), but the protein-centric F-max of the two methods are equal (0.20) (Fig. 2). This suggests that the choice of performance metric can have an impact on the rankings between methods on a particular task. The shortcomings of protein-centric measures are well known, namely the over-emphasis on accuracy for GO categories that have many annotations, that are less specific and that are not as informative. However, the limited time-frame for acquiring new annotations for a CAFA-like competition precludes the use of term-centric measures unless a large number of annotations are obtained for very specific functions. In our experiments we used a 5-year time frame for acquiring new annotations, whereas the CAFA experiment had only a few months [29].

## Discussion

Our study has multiple implications for the field of AFP. Our results demonstrate that the two AFP subtasks of making predictions on annotated proteins and previously unannotated proteins are much more difficult than performing well in CV, especially the task of predicting annotations for already annotated proteins.

This suggests that the task of predicting annotations for already annotated proteins could benefit from algorithms that explicitly use existing annotations to better rank novel predicted annotations. This can be accomplished in various ways, one of the simplest of which is to use the existing annotations as features and extending them e.g. using a nearest neighbor approach (see e.g. [30]). It is worth exploring how both existing annotations and other data can be used together for this task; with methods such as GOstruct and label-reconciliation methods such as described in Guan et al. [31], information on existing annotations can be encoded in the inference procedure itself.

Another important observation is that different methods are affected differently by the difficulty of the two AFP tasks, leading to different rankings of AFP methods under different evaluation protocols. For AFP practitioners this implies that using CV is not a good proxy for the performance in the more realistic AFP tasks, an observation that should be taken into account in performing model selection. Our focus in this study has been on methods that perform function prediction in a given species, and it remains to be seen to what extent our observations apply to nearest-neighbor type homology-based methods that are also commonly used in this field.

Finally, we observed that different performance measures can lead to different rankings of AFP methods, and more specifically, a difference when comparing protein-centric and term-centric performance. Although we recognize that in a CAFA-like competition it is not realistic to use term-centric performance, our results should contribute to the ongoing conversation in the AFP community on the appropriate way to evaluate AFP methods, suggesting that the use of multiple evaluation measures is necessary to accurately compare AFP methods.

## Methods

Each AFP method provides a confidence score for each of its predictions. Following the same procedure that was used in CAFA [5] these scores are recursively propagated upwards towards the root by assigning the highest score among children to their parent term. The true path rule is also applied to the ground truth in all three setups. However, in the NA setup, the older annotations (i.e. annotations acquired before 2009) are removed from the ground truth used for testing. By using a range of thresholds on these propagated confidence scores we compute the following protein-centric and term-centric measures.

In what follows *N* denotes the number of proteins and *M* is the number of GO categories. Protein-centric precision and recall at a threshold *t* are defined as

$$P^{pc}(t)=\frac{1}{N} \sum\limits_{i=0}^{N} \frac{TP_{i}(t)}{TP_{i}(t) + FP_{i}(t)}, $$

$$R^{pc}(t)=\frac{1}{N} \sum\limits_{i=0}^{N} \frac{TP_{i}(t)}{TP_{i}(t) + FN_{i}(t)}, $$

where *TP*_*i*_(*t*), *FP*_*i*_(*t*) and *FN*_*i*_(*t*) are the number of true positives, number of false positives and number of false negatives with respect to protein *i* at threshold *t*. Now we can define protein-centric F-max as

$$F\text{-}{max}^{pc} = \max_{t} \frac{2P^{pc}(t) R^{pc}(t)}{ P^{pc}(t)+ R^{pc}(t)}. $$

Precision and recall for a GO category *j* are defined as

$$P_{j}(t)= \frac{TP_{j}(t)}{TP_{j}(t) + FP_{j}(t)}, $$

$$R_{j}(t)= \frac{TP_{j}(t)}{TP_{j}(t) + FN_{j}(t)}, $$

where *TP*_*j*_(*t*), *FP*_*j*_(*t*) and *FN*_*j*_(*t*) are the number of true positives, number of false positives and number of false negatives with respect to GO category *j* at threshold *t*, respectively. Then, F-max for GO category *j* is defined as

$$F\text{-}{max}_{j}=\max_{t} \frac{2 P_{j}(t) R_{j}(t)}{P_{j}(t)+R_{j}(t)}\,. $$

An overall term-centric *F*-*max* (*F*-*max*^*tc*^) is obtained by finding the mean of the above individual values.

### Features

Each method was trained and tested using the same set of features and labels, prepared as described below.

#### GO annotations

We extracted GO annotations from the Gene Ontology website [11] and Uniprot-goa [12]. We ignored the root categories of the three subontologies. We also ignored the category ‘protein-binding’, which is uninformative, as also done in the CAFA1 challenge. We excluded all annotations that were obtained by computational methods, and we also did not include GO annotations with evidence codes that suggest that the annotation was derived from an interaction assay (i.e. only the evidence codes EXP, IDA, IMP, IGI, IEP and TAS were included). We also removed GO categories with fewer than 10 proteins were annotated.

#### Trans/Loc

We generated three sets of features using amino acid sequence properties: localization features, transmembrane features and low-complexity features described elsewhere [13]. The localization of a protein can be informative about its functions because many biological processes are known to be localized to certain cellular compartments [14]. We used the localization signals computed from the WOLF PSORT program [15] as localization features. The number of transmembrane domains a protein has can be informative of function. For example, transmembrane proteins are known to be associated with functions that involve transport of various molecules. We used TMHMM [16] to predict the number of transmembrane domains of each protein and this number was associated with an indicator variable. Low complexity regions are known to have an effect on protein function [17]. We used a sliding window approach (window length = 20) to identify the region with the lowest number of distinct amino acids and used the composition of that region as the representation of that region.

#### Homology

Homology to annotated proteins in other species was captured using an approach similar to GOtcha scores, as suggested in [3], and also used successfully by Radivojac’s team in the first CAFA competition [5]. Each protein is characterized by a feature vector where the *j*th feature is a confidence score that the protein is similar to proteins that are annotated with the *j*th GO category. Let *S*_*j*_ be the set of all sequences annotated with GO category *j* and let *e*(*s*,*s*^′^) be the e-value reported by BLAST+ [18] for the alignment of sequences *s* and *s*^′^ when querying *s* against the database containing *s*^′^. We define the e-max score for protein *s* and GO category *j* as:

$$e\text{-}max_{j}(s) = \max_{s' \in S_{j}} (-log(e(s,s')/10)). $$

This is a simpler version of *GOtcha* scores, observed to perform slightly better in preliminary experiments. The e-max scores efficiently integrate evidence for a given GO category across multiple species, and a protein is represented as a vector of variables where component *j* provides the e-max-score for GO category *j*. When running BLAST+ we used *psiblast* with one iteration on a database composed of all annotated sequences from every species except the target species. For example, when running BLAST+ for computing e-max-scores for yeast, the query consisted of all annotated yeast sequences and the database was composed of the annotated sequences from rest of the species. This approach ensures that annotations of the test sequences are not used by the classifier during the training phase. Additionally, we filtered out low complexity regions using the built-in *SEG* filter.

#### Network

We extracted protein-protein interactions and other functional association network data (protein-protein interactions, co-expression, protein co-occurrence, etc.) from BioGRID 3.2.106 [19], STRING 9.1 [20] and GeneMANIA 3.1.2 [21] in two species: human and yeast. The BioGRID database provides protein-protein interaction networks acquired from physical and genetic interaction experiments. STRING provides networks based on several different evidence channels (co-expression, co-occurrence, fusion, neighborhood, genetic interactions, physical interactions, etc.). For a given type of functional association data we combined edges from the two databases by taking the union of interactions from BioGRID and STRING and represented each gene by a vector of variables, where component *i* indicates if the corresponding protein interacts with protein *i* in the combined network. The GeneMANIA website provides a large number of protein-protein interaction and association networks generated using several types of evidence: co-expression, co-localization, genetic interactions, physical interactions and predicted interactions. A gene is represented by a vector of variables for each network, where component *i* indicates if the corresponding protein interacts with protein *i* with respect to that particular network.

#### Literature

If a protein is mentioned in the vicinity of a functional category in the literature (a co-mention) it is likely that this protein may be associated with that function. To make use of this source of information we extracted protein-functional category co-mentions using the natural language processing pipeline described in [22] from all Medline abstracts available on 23 October 2013 and full-text articles available from the PubMed Open Access Collection (PMCOA) on 06 November 2013.

The co-mentions we considered are the pairs of protein name and GO category mentioned in the document within a specified span. We used two spans: sentence and non-sentence. Sentence co-mentions only consider proteins and GO categories mentioned in the same sentence, whereas non-sentence co-mentions consider such pairs mentioned together in the same paragraph or abstract but not within the same sentence.

We provided the abstracts and full-text documents (one paragraph at a time) as input to our text mining pipeline. It detected protein entities in the given text and mapped them to UniProt identifiers through a specially constructed dictionary. This dictionary consists of all yeast and human target proteins from CAFA2. Similarly, another dictionary based on GO categories available on 13 November 2013 is used to recognize GO categories in the text. Finally the pipeline outputs the protein names and GO categories along with the counts of how often they appear together within the sentence or non-sentence spans.

A protein is represented by two vectors in which the *i*th element in each vector gives the number of times that protein is co-mentioned with the GO category *i* within either a sentence or non-sentence span. The vectors are concatenated to form the overall representation.

### Models

We used following three AFP approaches in our experiments: GOstruct [4], a collection of binary SVMs, and a network-based guilt-by-association method (GBA).

#### GOstruct

GOstruct [4] is an AFP method that uses a structured SVM [23], which allows it to explicitly model GO term prediction as a hierarchical multi-label prediction problem. The structured SVM can address prediction problems in which the labels, or outputs, have complex inter-relationships; in the AFP setup, this allows us to use a single classifier for each of the GO subontologies. Like other structured-output methods, the GOstruct structured SVM learns a compatibility function that models the association between a given input and a structured output [23], as described next. Let $\mathcal {X}$ be the input space where proteins are represented and let $\mathcal {Y}$ be the space of labels (GO categories). The set of GO categories annotated to a given gene is collectively referred to as its (structured) label. $\mathcal {Y}$ represents each GO subontology in a vector space where component *i* represents category *i*. Given a training set { (*x*_*i*_,*y*_*i*_)}$_{i=1}^{n}$ where $x_{i}\epsilon \mathcal {X} \quad \text {and} \quad y_{i}\epsilon \mathcal {Y}$, the compatibility function $f: \mathcal {X}\times \mathcal {Y}\mapsto \mathbb {R}$ maps input-output pairs to a score that indicates the strength of the association of an input to a set of GO categories. The predicted label $\hat {y}$ for an input *x* can then be obtained using the argmax operator as $\hat {y}=\arg \max _{y \in \mathcal {Y}_{c}} f(x,y) $ where $\mathcal {Y}_{c}\subset \mathcal {Y}$ is the set of all candidate labels. In this work we use the combinations of all categories in the training set as the set of candidate labels $\mathcal {Y}_{c}$.

To obtain correct classification, the compatibility value of the true label (correct set of GO annotations) of an input needs to be higher than that of any other candidate label. GOstruct uses structured SVM training in which this is used as a soft constraint; it tries to maximize the margin, or the difference between the compatibility value for the actual label and the compatibility for the next best candidate [23]. In the structured-output setting, kernels correspond to dot products in the joint input-output feature space, and they are functions of both inputs and outputs. GOstruct uses a joint kernel that is the product of the input-space and the output-space kernels:

$$K((x_{1}, y_{1}), (x_{2}, y_{2})) = K_{\mathcal{X}}(x_{1}, x_{2})K_{\mathcal{Y}}(y_{1}, y_{2}). $$

Different sources of data are combined by adding linear kernels at the input-space level, and for the output space we use a linear kernel between label vectors. Each kernel is normalized according to

$$K_{norm}(z_{1}, z_{2}) = K(z_{1}, z_{2})/\sqrt{K(z_{1}, z_{1})K(z_{2}, z_{2})} $$

before being used to construct the joint input-output kernel. The Strut library [24] with default parameter settings was used for running GOstruct.

#### Binary SVMs

We trained a collection of binary SVMs, each trained on a single GO category. Binary SVMs were trained using the PyML [25] machine learning library with default parameter settings. The SVMs used the same input-space kernels as GOstruct.

#### GBA

GBA is the in-house Python implementation of the simplest form of guilt-by-association [26] AFP algorithm (i.e. neighbor-voting) also called the Basic GBA (BGBA) [27]. It is a binary classifier that computes a confidence score with respect to a given test protein and a GO category by using the connectivity of the test protein to other proteins annotated with that GO category in a given input network. More specifically, this score is equal to the fraction of direct neighbors that are annotated with that category. The kernel used for the SVM methods was used as a network for the GBA procedure.

## Availability of supporting data

The data sets supporting the results of this article are available in the GigaScience GigaDB repository, [32]. This contains all the input data (both features and labels) and predictions from the three methods (GOstruct, SVM and GBA).

## Additional file

Additional file 1 **Supplementary material.** All the tables and figures in this file are listed below. **Table S1.** Difference in performance between CV and NA/NP for yeast. **Table S2.** Difference in performance between CV and NA/NP for human. **Figure S1.** Performance comparison between CV, NA and NP in molecular function subontology for yeast and human. **Figure S2.** Performance comparison between CV, NA and NP in biological process subontology for yeast and human. **Figure S3.** Performance comparison between CV, NA and NP in cellular component subontology for yeast and human. **Figure S4.** Signed discrepancy (between probability of each GO category in train and test sets) for molecular function subontology of yeast. **Figure S5.** Signed discrepancy (between probability of each GO category in train and test sets) for biological process subontology of yeast. **Figure S6.** Signed discrepancy (between probability of each GO category in train and test sets) for cellular component subontology of yeast. **Figure S7.** Pearson correlation coefficient between discrepancy of each GO category and its individual AUC. **Table S3.** Pearson correlation coefficient between the performance of the node degree classifier (NDC) of each GO category and its individual AUC. **Figure S8.** Pearson correlation coefficient between discrepancy of each GO category and its individual AUC obtained by the NDC classifier. **Table S4.** Performance comparison between CV, NA and NP in all three subontologies for yeast for well-represented GO categories. **Table S5.** Performance comparison between CV, NA and NP in all three subontologies for human for well-represented GO categories. **Figure S9.** Comparison between the NA and NP evaluation protocols. **Table S6.** Performance comparison between CV, NA and NP in all three subontologies for yeast. **Table S7.** Performance comparison between CV, NA and NP in all three subontologies for human. (PDF 590 kb)
