# Supplementary material for: A close look at protein function prediction evaluation protocols
Source: Gigascience. 2015 Sep 14;4:41. doi: 10.1186/s13742-015-0082-5 (PMC4570743; doi:10.1186/s13742-015-0082-5)
Supplement: Supplementary file 1 — Supplementary material. All the tables and figures in this file are listed below. Table S1. Difference in performance between CV and NA/NP for yeast. Table S2. Difference in performance between CV and NA/NP for human. Figure S1. Performance comparison between CV, NA and NP in molecular function subontology for yeast and human. Figure S2. Performance comparison between CV, NA and NP in biological process subontology for yeast and human. Figure S3. Performance comparison between CV, NA and NP in cellular component subontology for yeast and human. Figure S4. Signed discrepancy (between probability of each GO category in train and test sets) for molecular function subontology of yeast. Figure S5. Signed discrepancy (between probability of each GO category in train and test sets) for biological process subontology of yeast. Figure S6. Signed discrepancy (between probability of each GO category in train and test sets) for cellular component subontology of yeast. Figure S7. Pearson correlation coefficient between discrepancy of each GO category and its individual AUC. Table S3. Pearson correlation coefficient between the performance of the node degree classifier (NDC) of each GO category and its individual AUC. Figure S8. Pearson correlation coefficient between discrepancy of each GO category and its individual AUC obtained by the NDC classifier. Table S4. Performance comparison between CV, NA and NP in all three subontologies for yeast for well-represented GO categories. Table S5. Performance comparison between CV, NA and NP in all three subontologies for human for well-represented GO categories. Figure S9. Comparison between the NA and NP evaluation protocols. Table S6. Performance comparison between CV, NA and NP in all three subontologies for yeast. Table S7. Performance comparison between CV, NA and NP in all three subontologies for human. (PDF 590 kb) [file 13742_2015_82_MOESM1_ESM.pdf]

A close look at automated protein function prediction evaluation protocols:

Supplementary material

Table S1: Difference in performance ($F\text{-max}^{pc}$) between CV and NA/NP for yeast and the corresponding p-values computed using paired t-tests. F, P and C represent molecular function, biological process and cellular component, respectively. P-values < 0.05 are in bold.

Subont.	Method	CV	NA	p-value	NP	p-value
**F**	**GOstruct**	0.73	0.33	**1.46 x 10-61**	0.71	2.15 x 10-01
	**Binary SVMs**	0.53	0.18	**8.05 x 10-14**	0.25	**5.85 x 10-31**
	**GBA**	0.54	0.21	**1.84 x 10-18**	0.37	**5.69 x 10-21**
**P**	**GOstruct**	0.70	0.29	**1.07 x 10-158**	0.68	7.68 x 10-02
	**Binary SVMs**	0.53	0.17	**4.85 x 10-117**	0.30	**9.49 x 10-36**
	**GBA**	0.56	0.28	**2.97 x 10-151**	0.46	**3.30 x 10-23**
**C**	**GOstruct**	0.80	0.47	**6.35 x 10-263**	0.81	3.25 x 10-01
	**Binary SVMs**	0.50	0.28	**6.00 x 10-212**	0.49	2.31 x 10-01
	**GBA**	0.70	0.48	**4.13 x 10-267**	0.60	**3.03 x 10-10**

Table S2: Difference in performance ($F\text{-max}^{pc}$) between CV and NA/NP for human and the corresponding p-values computed using paired t-tests. F, P and C represent molecular function, biological process and cellular component, respectively. P-values < 0.05 are in bold.

Subont.	Method	CV	NA	p-value	NP	p-value
**F**	GOstruct	0.60	0.26	**3.32 x 10-167**	0.57	**6.38 x 10-04**
	Binary SVMs	0.55	0.20	**1.20 x 10-150**	0.39	**3.82 x 10-16**
	GBA	0.41	0.20	**2.97 x 10-114**	0.34	**1.03 x 10-18**
**P**	GOstruct	0.64	0.24	**< 1 x 10-308**	0.55	**3.83 x 10-67**
	Binary SVMs	0.56	0.23	**< 1 x 10-308**	0.38	**4.97 x 10-212**
	GBA	0.52	0.27	**< 1 x 10-308**	0.37	**3.15 x 10-260**
**C**	GOstruct	0.72	0.34	**< 1 x 10-308**	0.67	**3.08 x 10-14**
	Binary SVMs	0.50	0.21	**< 1 x 10-308**	0.39	**3.58 x 10-107**
	GBA	0.60	0.36	**< 1 x 10-308**	0.45	**< 1 x 10-308**

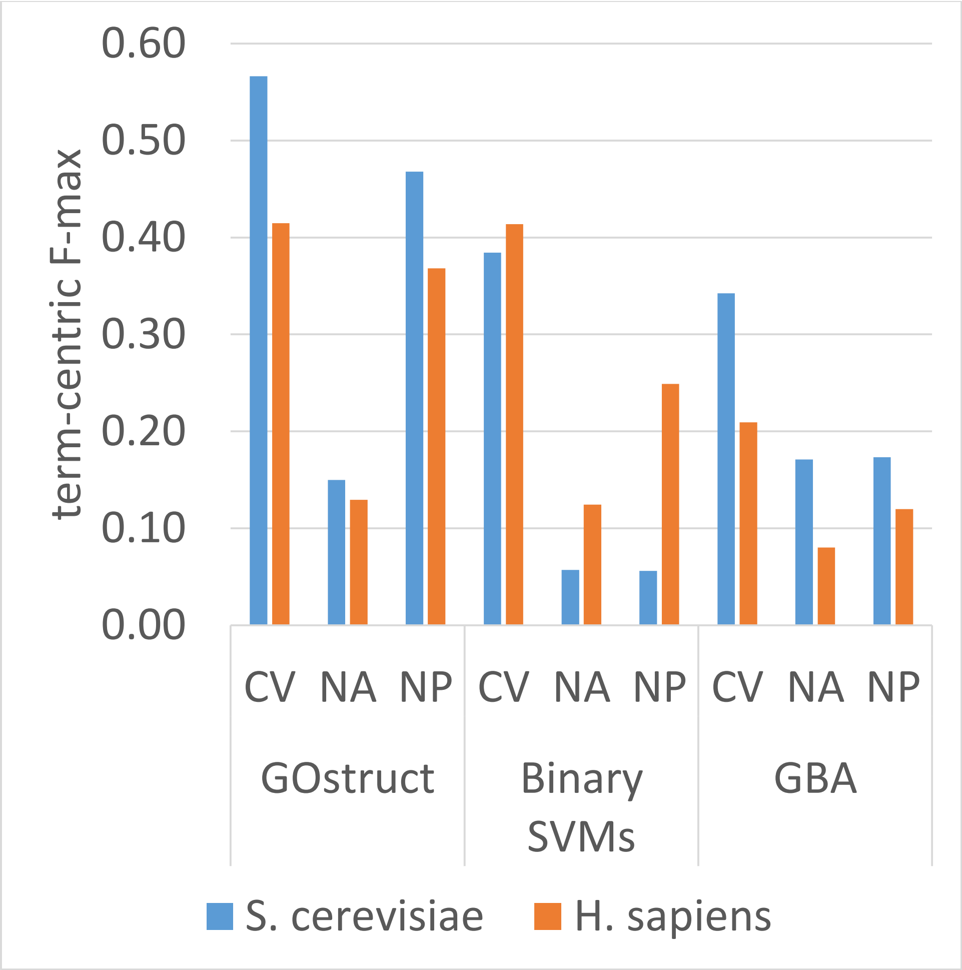

Figure S1: Performance comparison between CV, NA and NP in molecular function subontology. GOstruct, Binary SVMs and GBA are evaluated in CV (cross-validation), NA (novel-annotation) and NP (novel-proteins) in yeast and human. Performance is presented in term-centric F-max.

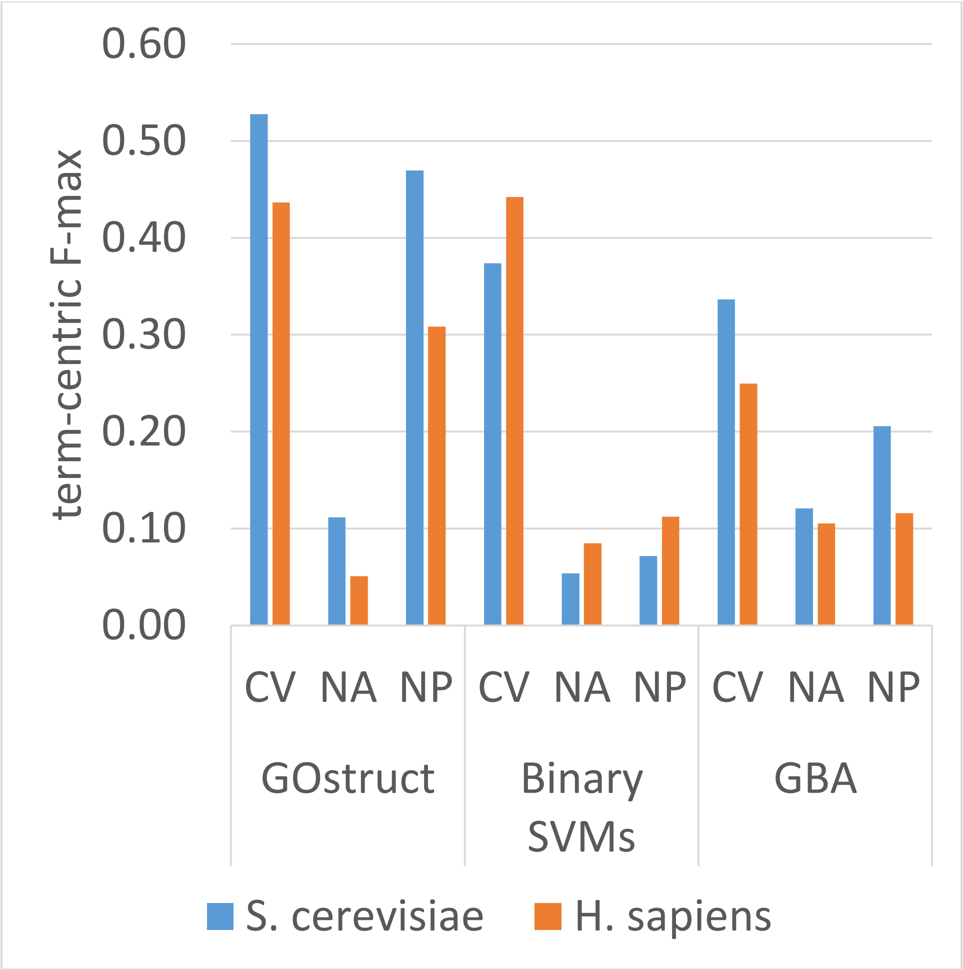

Figure S2: Performance comparison between CV, NA and NP in biological process subontology. GOstruct, Binary SVMs and GBA are evaluated in CV (cross-validation), NA (novel-annotation) and NP (novel-proteins) in yeast and human. Performance is presented in term-centric F-max.

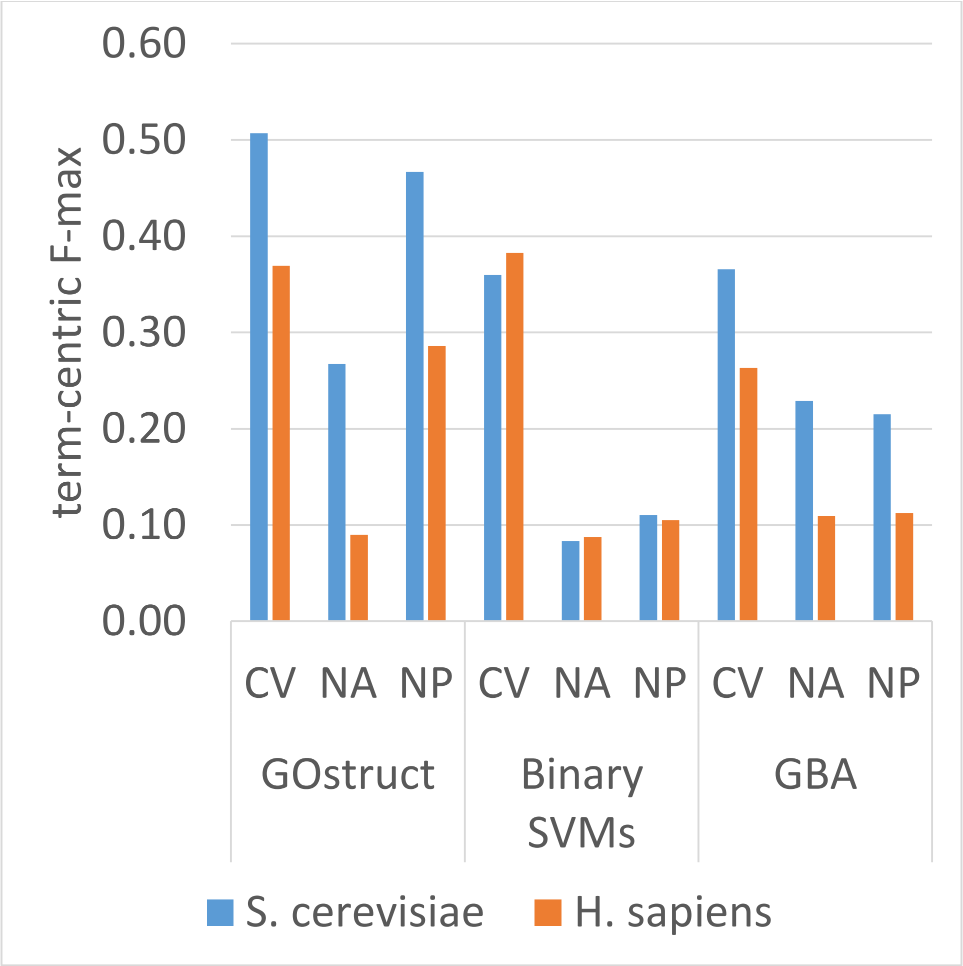

Figure S3: Performance comparison between CV, NA and NP in cellular component subontology. GOstruct, Binary SVMs and GBA are evaluated on CV (cross-validation), NA (novel-annotation) and NP (novel-proteins) in yeast and human. Performance is presented in term-centric F-max.

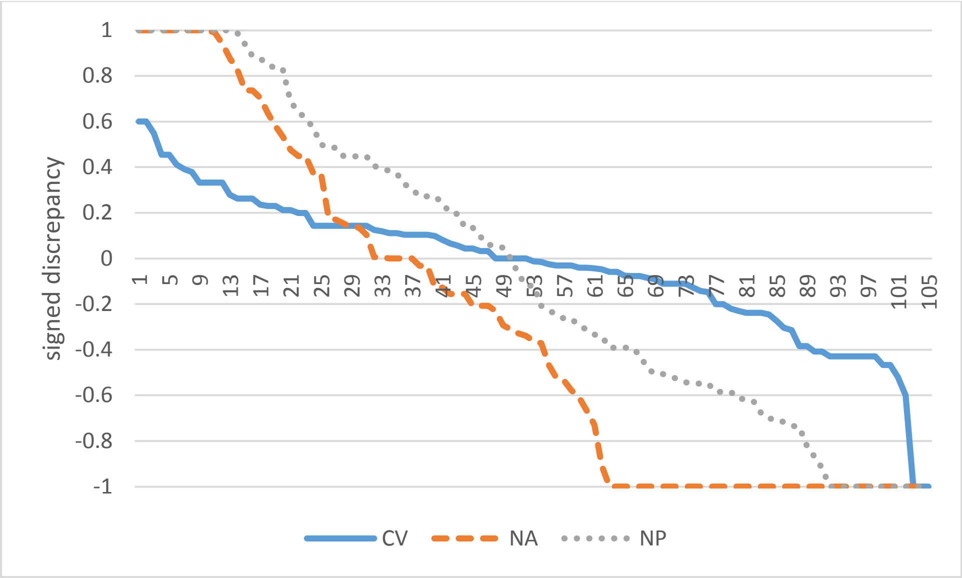

Figure S4: Signed discrepancy (between probability of each GO category in train and test sets) comparison between CV, NA and NP for molecular function subontology of yeast. Y-axis gives the indices of the GO categories. GO categories are ordered in the descending order of the signed discrepancy within each setup (CV, NA and NP).

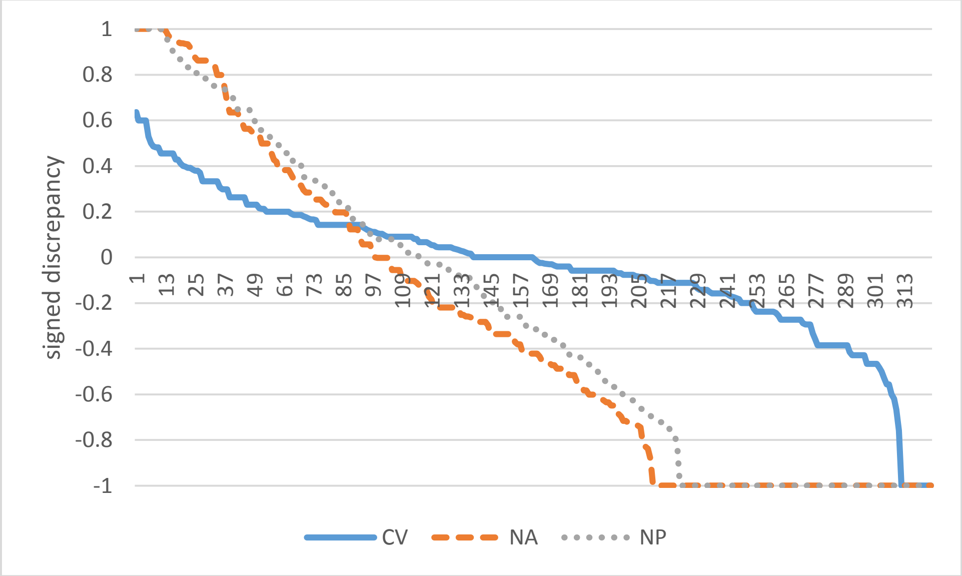

Figure S5: Signed discrepancy (between probability of each GO category in train and test sets) comparison between CV, NA and NP for biological process subontology of yeast. Y-axis gives the indices of the GO categories. GO categories are ordered in the descending order of the signed discrepancy within each setup (CV, NA and NP).

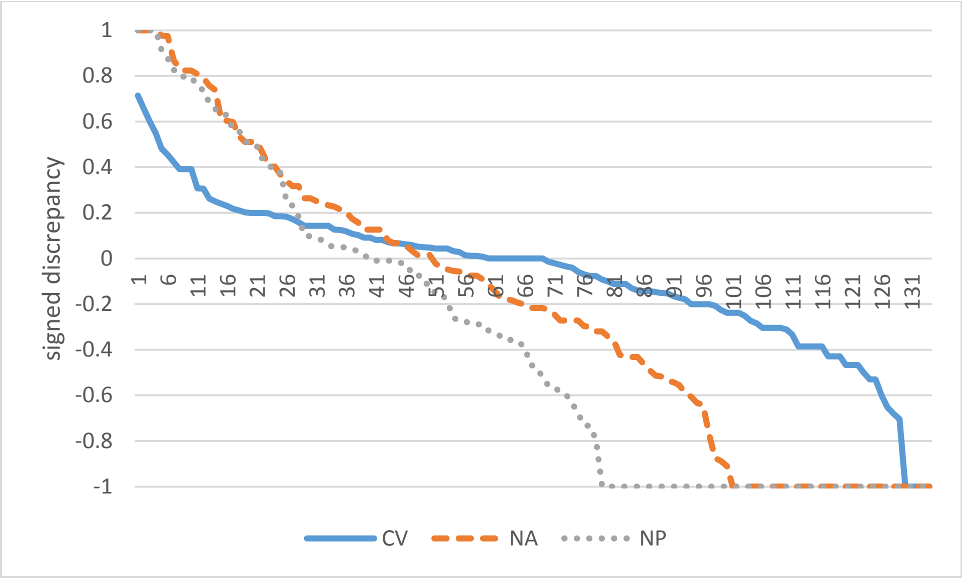

Figure S6: Signed discrepancy (between probability of each GO category in train and test sets) comparison between CV, NA and NP for cellular component subontology of yeast. Y-axis gives the indices of the GO categories. GO categories are ordered in the descending order of the signed discrepancy within each setup (CV, NA and NP).

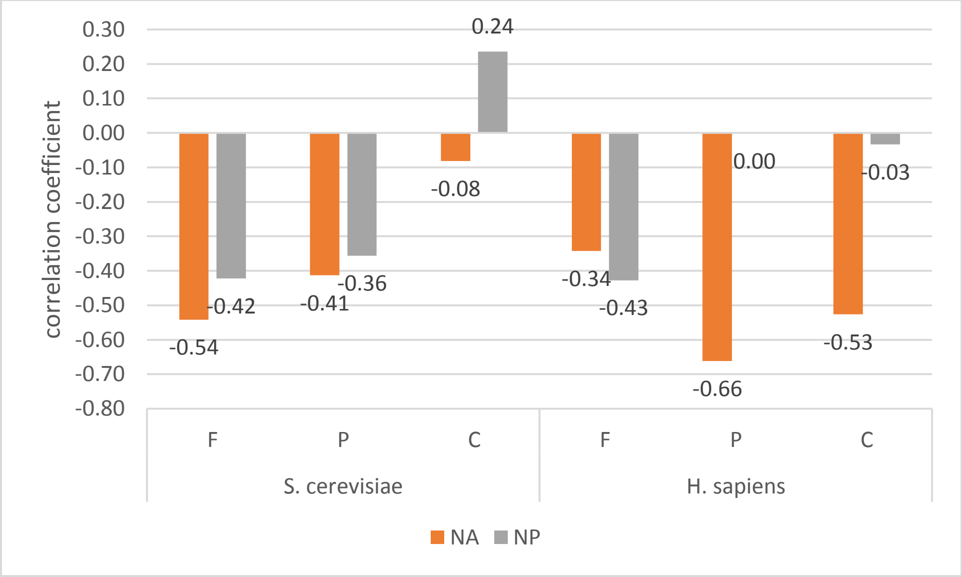

Figure S7: Pearson correlation coefficient between discrepancy of each GO category and its individual AUC. P-values for NA and NP of S. cerevisiae are 2.33 x 10-13 and 3.67 x 10-08 (molecular function), 1.19 x 10-29 and 5.81 x 10-22 (biological process) and 2.88 x 10-01 and 1.87 x 10-03 (cellular component). Corresponding p-values for H. sapiens are 7.94 x 10-11 and 1.40 x 10-16 (molecular function), 5.09 x 10-228 and 9.26 x 10-01 (biological process) and 1.18 x 10-22 and 5.65 x 10-01 (cellular component).

Table S3: Pearson correlation coefficient between the performance of the node degree classifier (NDC) of each GO category and its individual AUC computed using other methods in the biological process subontology of yeast and human.

Species	method	setup	correlation	p-value
**S. cerevisiae**	**GOstruct**	**CV**	0.20	**2.00 x 10-07**
		**NA**	0.09	**2.05 x 10-02**
		**NP**	0.21	**3.76 x 10-08**
	**Binary SVMs**	**CV**	0.29	**1.22 x 10-13**
		**NA**	-0.03	5.20 x 10-01
		**NP**	0.06	1.56 x 10-01
	**GBA**	**CV**	0.14	**2.51 x 10-04**
		**NA**	0.03	4.74 x 10-01
		**NP**	0.18	**2.18 x 10-06**
**H. sapiens**	**GOstruct**	**CV**	0.44	**3.98 x 10-88**
		**NA**	-0.11	**4.77 x 10-06**
		**NP**	0.28	**1.62 x 10-34**
	**Binary SVMs**	**CV**	0.34	**7.83 x 10-51**
		**NA**	0.00	9.42 x 10-01
		**NP**	0.06	**9.09 x 10-03**
	**GBA**	**CV**	0.33	**3.05 x 10-46**
		**NA**	0.07	**2.22 x 10-03**
		**NP**	0.16	**2.21 x 10-11**

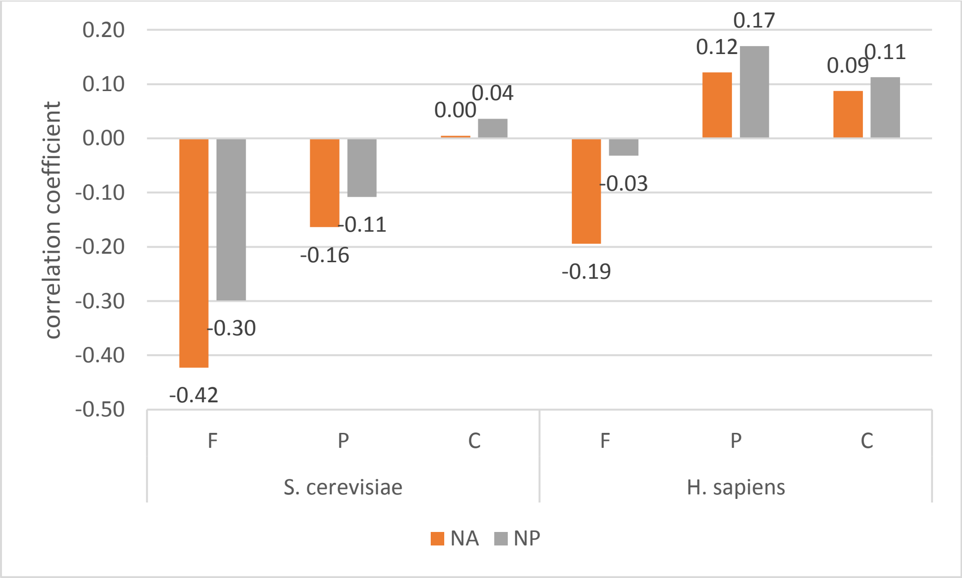

Figure S8: Pearson correlation coefficient between discrepancy of each GO category and its individual AUC obtained by the NDC classifier. P-values for NA and NP of S. cerevisiae are 3.52 x 10-08 and 1.45 x 10-04 (molecular function), 1.63 x 10-05 and 4.62 x 10-03 (biological process) and 9.48 x 10-01 and 6.37 x 10-01 (cellular component). Corresponding p-values for H. sapiens are 3.14 x 10-04 and 5.58 x 10-01 (molecular function), 2.12 x 10-07 and 3.07 x 10-13 (biological process) and 1.32 x 10-01 and 5.22 x 10-02 (cellular component).

Table S4: Performance comparison between CV, NA and NP in all three subontologies. GOstruct, Binary SVMs and GBA are evaluated on CV (cross-validation), NA (novel-annotation) and NP (novel-proteins) in yeast for well-represented GO categories (# annotations >= 50. for yeast for well-represented GO categories (# annotations >= 50. Performance is presented in both protein-centric and term-centric measures.

subont.	method	setup	F-max^{pc}	P^{pc}	R^{pc}	AUC^{tc}	F-max^{tc}	P^{tc}	R^{tc}
F	GOstruct	CV	0.81	0.79	0.83	0.94	0.74	0.79	0.73
F	GOstruct	NA	0.54	0.45	0.72	0.87	0.14	0.08	1.00
F	GOstruct	NP	0.78	0.77	0.79	0.94	0.73	0.71	0.77
F	Binary SVMs	CV	0.62	0.56	0.69	0.89	0.58	0.58	0.59
F	Binary SVMs	NA	0.42	0.33	0.58	0.71	0.13	0.08	0.43
F	Binary SVMs	NP	0.32	0.25	0.44	0.58	0.18	0.11	0.77
F	GBA	CV	0.61	0.56	0.68	0.86	0.53	0.49	0.64
F	GBA	NA	0.56	0.59	0.54	0.79	0.26	0.28	0.31
F	GBA	NP	0.48	0.42	0.56	0.73	0.35	0.31	0.50
P	GOstruct	CV	0.72	0.69	0.75	0.92	0.61	0.60	0.64
P	GOstruct	NA	0.34	0.30	0.42	0.80	0.22	0.18	0.50
P	GOstruct	NP	0.69	0.62	0.80	0.91	0.59	0.57	0.63
P	Binary SVMs	CV	0.57	0.53	0.61	0.87	0.45	0.42	0.54
P	Binary SVMs	NA	0.21	0.14	0.52	0.58	0.10	0.06	1.00
P	Binary SVMs	NP	0.33	0.24	0.54	0.59	0.15	0.09	0.87
P	GBA	CV	0.59	0.57	0.61	0.87	0.46	0.43	0.59
P	GBA	NA	0.33	0.27	0.43	0.78	0.22	0.22	0.32
P	GBA	NP	0.48	0.42	0.57	0.80	0.31	0.28	0.52
C	GOstruct	CV	0.81	0.79	0.82	0.90	0.57	0.60	0.59
C	GOstruct	NA	0.49	0.44	0.56	0.86	0.31	0.27	0.48
C	GOstruct	NP	0.82	0.80	0.84	0.90	0.55	0.56	0.58
C	Binary SVMs	CV	0.55	0.50	0.61	0.86	0.41	0.46	0.47
C	Binary SVMs	NA	0.32	0.27	0.41	0.71	0.17	0.12	0.64
C	Binary SVMs	NP	0.53	0.42	0.72	0.66	0.18	0.14	0.55
C	GBA	CV	0.72	0.69	0.74	0.85	0.43	0.40	0.56
C	GBA	NA	0.50	0.42	0.62	0.84	0.28	0.23	0.56
C	GBA	NP	0.62	0.56	0.68	0.77	0.25	0.20	0.53

Table S5: Performance comparison between CV, NA and NP in all three subontologies. GOstruct, Binary SVMs and GBA are evaluated on CV (cross-validation), NA (novel-annotation) and NP (novel-proteins) in human for well-represented GO categories (# annotations >= 50). F, P and C represent molecular function, biological process and cellular component, respectively. Performance is presented in both protein-centric and term-centric measures.

subont.	method	setup	F-max^{pc}	P^{pc}	R^{pc}	AUC^{tc}	F-max^{tc}	P^{tc}	R^{tc}
F	GOstruct	CV	0.63	0.60	0.66	0.89	0.56	0.57	0.57
		NA	0.30	0.25	0.41	0.82	0.18	0.12	0.67
		NP	0.59	0.56	0.63	0.89	0.53	0.60	0.51
	Binary SVMs	CV	0.59	0.58	0.60	0.90	0.52	0.54	0.55
		NA	0.25	0.23	0.27	0.82	0.18	0.17	0.41
		NP	0.45	0.40	0.50	0.85	0.35	0.35	0.45
	GBA	CV	0.44	0.43	0.45	0.75	0.28	0.22	0.43
		NA	0.24	0.20	0.31	0.67	0.12	0.09	0.31
		NP	0.40	0.51	0.34	0.65	0.17	0.13	0.32
P	GOstruct	CV	0.66	0.62	0.71	0.87	0.52	0.55	0.50
		NA	0.27	0.23	0.35	0.68	0.16	0.14	0.26
		NP	0.57	0.49	0.68	0.84	0.40	0.43	0.41
	Binary SVMs	CV	0.59	0.58	0.60	0.87	0.50	0.53	0.51
		NA	0.29	0.24	0.36	0.71	0.17	0.16	0.26
		NP	0.42	0.39	0.46	0.73	0.23	0.25	0.27
	GBA	CV	0.55	0.57	0.52	0.81	0.35	0.31	0.44
		NA	0.31	0.28	0.34	0.68	0.18	0.13	0.49
		NP	0.40	0.40	0.40	0.66	0.20	0.17	0.32
C	GOstruct	CV	0.72	0.70	0.75	0.85	0.48	0.55	0.46
		NA	0.36	0.32	0.43	0.73	0.18	0.15	0.36
		NP	0.68	0.62	0.74	0.84	0.41	0.46	0.43
	Binary SVMs	CV	0.55	0.48	0.64	0.85	0.41	0.48	0.45
		NA	0.26	0.20	0.36	0.75	0.17	0.16	0.32
		NP	0.43	0.34	0.59	0.73	0.19	0.19	0.34
	GBA	CV	0.61	0.58	0.64	0.77	0.35	0.33	0.42
		NA	0.37	0.32	0.45	0.67	0.19	0.15	0.44
		NP	0.46	0.48	0.44	0.62	0.18	0.15	0.39

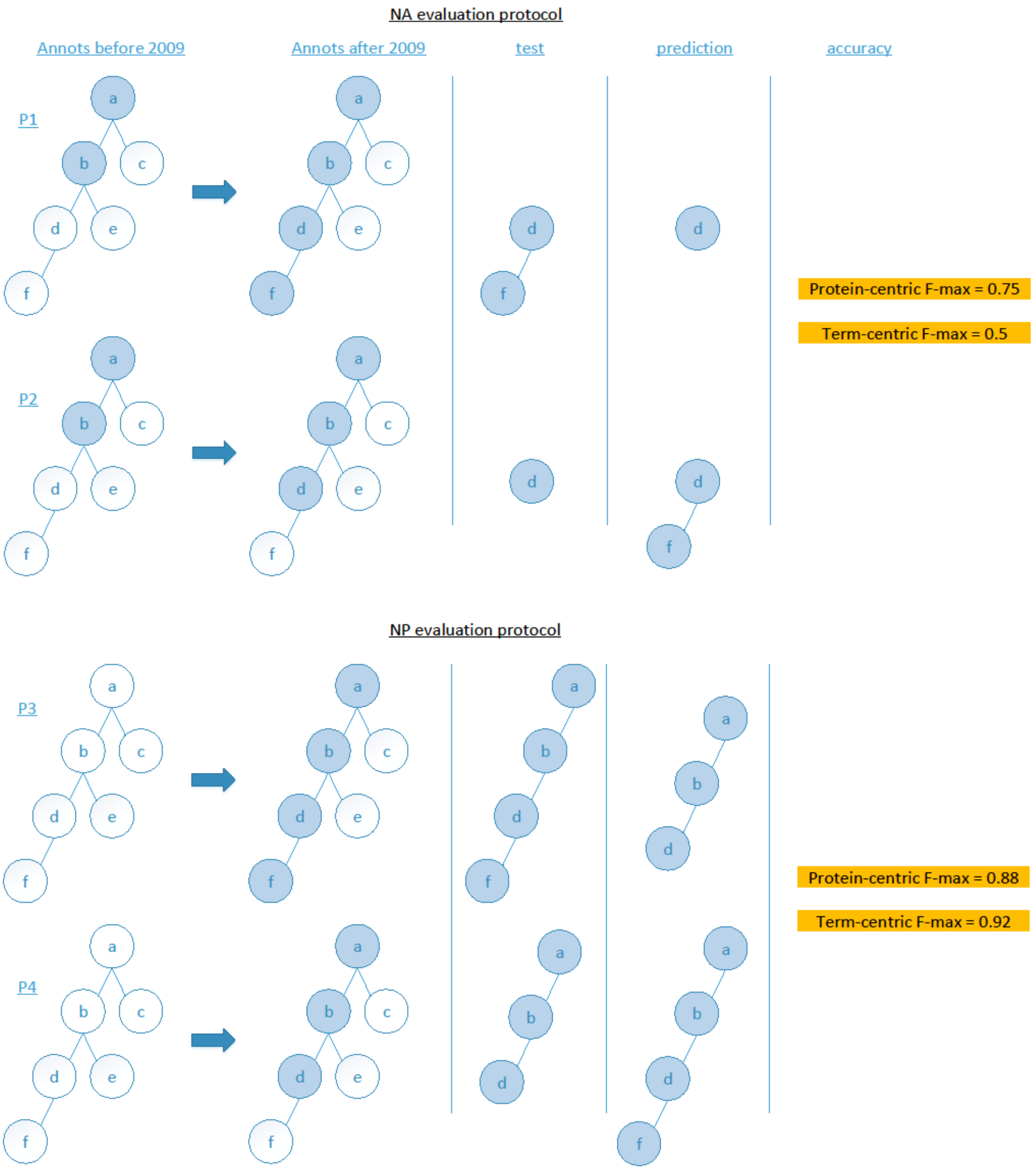

Figure S9: Comparison between the NA and NP evaluation protocols. Let P1, P2 and P3, P4 be two pairs of test proteins used for evaluating accuracy of NA and NP setups, respectively. The first two columns show the annotations acquired before and after 2009. The column "test" shows the ground truth used for testing and column "prediction" shows the prediction made by the classifier for each protein. Finally, the column "accuracy" shows the overall protein-centric and term-centric F-max values for each protocol based on the corresponding ground truths and predictions. Therefore, even if the classifier has made exactly the same predictions for P1 and P3 (i.e. d) as well as for P2 and P4 (i.e. f) both the evaluation measures gives lower values for NA protocol compared to NP.

Table S6: Performance comparison between CV, NA and NP in all three subontologies. GOstruct, Binary SVMs and GBA are evaluated on CV (cross-validation), NA (novel-annotation) and NP (novel-proteins) in yeast. F, P and C represent molecular function, biological process and cellular component, respectively. Performance is presented in both protein-centric and term-centric measures.

subont.	method	setup	F-max^{pc}	P^{pc}	R^{pc}	AUC^{tc}	F-max^{tc}	P^{tc}	R^{tc}
F	GOstruct	CV	0.73	0.72	0.75	0.93	0.57	0.58	0.59
		NA	0.33	0.28	0.43	0.80	0.15	0.09	0.69
		NP	0.71	0.71	0.72	0.91	0.47	0.46	0.50
	Binary SVMs	CV	0.53	0.52	0.54	0.88	0.38	0.36	0.46
		NA	0.18	0.13	0.26	0.65	0.06	0.04	0.31
		NP	0.25	0.20	0.32	0.56	0.06	0.03	0.54
	GBA	CV	0.54	0.55	0.54	0.85	0.34	0.31	0.44
		NA	0.21	0.23	0.19	0.70	0.17	0.17	0.25
		NP	0.37	0.37	0.38	0.69	0.17	0.19	0.24
P	GOstruct	CV	0.70	0.67	0.73	0.92	0.53	0.54	0.56
		NA	0.29	0.27	0.33	0.77	0.11	0.08	0.75
		NP	0.68	0.60	0.77	0.89	0.47	0.48	0.49
	Binary SVMs	CV	0.53	0.53	0.54	0.89	0.37	0.40	0.42
		NA	0.17	0.11	0.40	0.57	0.05	0.03	0.69
		NP	0.30	0.22	0.48	0.56	0.07	0.05	0.41
	GBA	CV	0.56	0.57	0.55	0.84	0.34	0.31	0.45
		NA	0.28	0.26	0.30	0.69	0.12	0.12	0.20
		NP	0.46	0.42	0.51	0.74	0.21	0.20	0.33
C	GOstruct	CV	0.80	0.78	0.81	0.93	0.51	0.57	0.51
		NA	0.47	0.44	0.51	0.89	0.27	0.25	0.40
		NP	0.81	0.79	0.83	0.92	0.47	0.51	0.49
	Binary SVMs	CV	0.50	0.47	0.54	0.91	0.36	0.42	0.43
		NA	0.28	0.22	0.40	0.68	0.08	0.06	0.55
		NP	0.49	0.41	0.61	0.66	0.11	0.08	0.53
	GBA	CV	0.70	0.69	0.71	0.86	0.37	0.32	0.50
		NA	0.48	0.41	0.58	0.81	0.23	0.19	0.52
		NP	0.60	0.56	0.65	0.80	0.21	0.19	0.40

Table S7: Performance comparison between CV, NA and NP in all three subontologies. GOstruct, Binary SVMs and GBA are evaluated on CV (cross-validation), NA (novel-annotation) and NP (novel-proteins) in human. F, P and C represent molecular function, biological process and cellular component, respectively. Performance is presented in both protein-centric and term-centric measures.

subont.	method	setup	F-max^{pc}	P^{pc}	R^{pc}	AUC^{tc}	F-max^{tc}	P^{tc}	R^{tc}
**F**	**GOstruct**	**CV**	0.60	0.58	0.62	0.88	0.41	0.43	0.44
		**NA**	0.26	0.23	0.30	0.73	0.13	0.09	0.44
		**NP**	0.57	0.49	0.69	0.85	0.37	0.43	0.35
	**Binary SVMs**	**CV**	0.55	0.55	0.55	0.89	0.41	0.41	0.47
		**NA**	0.20	0.22	0.19	0.79	0.12	0.11	0.39
		**NP**	0.39	0.35	0.44	0.78	0.25	0.24	0.34
	**GBA**	**CV**	0.41	0.43	0.39	0.73	0.21	0.18	0.29
		**NA**	0.20	0.17	0.22	0.62	0.08	0.07	0.17
		**NP**	0.34	0.50	0.26	0.61	0.12	0.10	0.20
**P**	**GOstruct**	**CV**	0.64	0.66	0.62	0.89	0.44	0.48	0.43
		**NA**	0.24	0.21	0.28	0.58	0.05	0.04	0.09
		**NP**	0.55	0.47	0.66	0.83	0.31	0.34	0.31
	**Binary SVMs**	**CV**	0.56	0.58	0.54	0.89	0.44	0.52	0.42
		**NA**	0.23	0.19	0.29	0.66	0.08	0.09	0.14
		**NP**	0.38	0.35	0.40	0.66	0.11	0.11	0.15
	**GBA**	**CV**	0.52	0.52	0.51	0.78	0.25	0.22	0.33
		**NA**	0.27	0.27	0.27	0.59	0.11	0.10	0.23
		**NP**	0.37	0.40	0.34	0.59	0.12	0.11	0.22
**C**	**GOstruct**	**CV**	0.72	0.70	0.73	0.85	0.37	0.45	0.36
		**NA**	0.34	0.31	0.39	0.66	0.09	0.07	0.21
		**NP**	0.67	0.62	0.73	0.83	0.29	0.29	0.33
	**Binary SVMs**	**CV**	0.50	0.44	0.57	0.87	0.38	0.51	0.38
		**NA**	0.21	0.15	0.35	0.70	0.09	0.10	0.19
		**NP**	0.39	0.32	0.52	0.69	0.10	0.14	0.17
	**GBA**	**CV**	0.60	0.58	0.62	0.77	0.26	0.21	0.42
		**NA**	0.36	0.30	0.43	0.63	0.11	0.10	0.23
		**NP**	0.45	0.48	0.43	0.62	0.11	0.10	0.29
